# Tumor laterality in renal cancer as a predictor of survival in large patient cohorts: A STROBE compliant study: Erratum

**DOI:** 10.1097/MD.0000000000015790

**Published:** 2019-05-17

In the article, “Tumor laterality in renal cancer as a predictor of survival in large patient cohorts: A STROBE compliant study”,^[1]^ which appears in Volume 98, Issue 17 or *Medicine*. The second sentence under 5. Conclusions is missing the word “not” and should read ““Those survival differences are predominantly observed among patients not undergoing LAD with left sided disease having worse CSS compared to patients with right sided tumor.”
